# Multivariate prediction of dementia in Parkinson’s disease

**DOI:** 10.1038/s41531-020-00121-2

**Published:** 2020-08-25

**Authors:** Thanaphong Phongpreecha, Brenna Cholerton, Ignacio F. Mata, Cyrus P. Zabetian, Kathleen L. Poston, Nima Aghaeepour, Lu Tian, Joseph F. Quinn, Kathryn A. Chung, Amie L. Hiller, Shu-Ching Hu, Karen L. Edwards, Thomas J. Montine

**Affiliations:** 1 Department of Anesthesiology, Perioperative and Pain Medicine, Stanford University School of Medicine, Palo Alto, CA USA; 2 Department of Pathology, Stanford University School of Medicine, Palo Alto, CA USA; 3 Department of Biomedical Data Science, Stanford University School of Medicine, Palo Alto, CA USA; 4 Genomic Medicine Institute, Cleveland Clinic, Cleveland, OH USA; 5 Veterans Affairs Puget Sound Health Care System, Seattle, WA USA; 6 Department of Neurology, University of Washington School of Medicine, Seattle, WA USA; 7 Department of Neurology and Neurological Sciences, Stanford School of Medicine, Palo Alto, CA USA; 8 Department of Pediatrics, Stanford University School of Medicine, Palo Alto, CA USA; 9 Portland Veterans Affairs Health Care System, Portland, OR USA; 10 Department of Neurology, Oregon Health and Science University, Portland, OR USA; 11 Department of Epidemiology, University of California, Irvine, School of Medicine, Irvine, CA USA

**Keywords:** Parkinson's disease, Dementia

## Abstract

Cognitive impairment in Parkinson’s disease (PD) is pervasive with potentially devastating effects. Identification of those at risk for cognitive decline is vital to identify and implement appropriate interventions. Robust multivariate approaches, including fixed-effect, mixed-effect, and multitask learning models, were used to study associations between biological, clinical, and cognitive factors and for predicting cognitive status longitudinally in a well-characterized prevalent PD cohort (*n* = 827). Age, disease duration, sex, and *GBA* status were the primary biological factors associated with cognitive status and progression to dementia. Specific cognitive tests were better predictors of subsequent cognitive status for cognitively unimpaired and dementia groups. However, these models could not accurately predict future mild cognitive impairment (PD-MCI). Data collected from a large PD cohort thus revealed the primary biological and cognitive factors associated with dementia, and provide clinicians with data to aid in the identification of risk for dementia. Sex differences and their potential relationship to genetic status are also discussed.

## Introduction

Cognitive impairment in Parkinson’s disease (PD) is pervasive with multiple negative effects^1^. The trajectory of cognitive decline in PD can vary considerably, however, with some individuals quickly developing cognitive symptoms that interfere with functional activities and others maintaining steady but mild symptoms over many years^2^. Because cognitive impairment can begin insidiously, such problems can go unrecognized and in the absence of appropriate behavioral, social, and medical interventions may interfere with patient safety and independence^3^. A current important question in PD research is thus whether those who are at risk for impending cognitive decline can be identified in order to implement appropriate interventions, optimize medical management, and enhance autonomy.

There is now abundant genetic and phenotypic data to support substantial clinical and biological heterogeneity in cognitive decline in people with PD, and this complexity challenges traditional methodological approaches^2,4^. There are thus many potential interactions between processes that underlie cognition and other biological systems among individuals that may introduce error. Conventional statistical approaches may thus result in poor reproducibility. Such methods are chosen by the researcher a priori and are used to test one or a few variables at a time, often with an overemphasis on *P* values and an inability to adequately address the potential impact of heterogeneity. Given these issues, the resulting conclusions may lack important clinical meaning and generalizability. In order to address the problems introduced by univariate statistical methods, multivariate models are used with increasing frequency in the study of cognitive diseases^5^.

Here, we utilized multivariate models, including fixed-effect, mixed-effect, and multitask learning models, to examine the interplay among cognition, genetics, and clinical features in the Pacific Udall Center (PUC), a large, deeply annotated cohort of participants with PD. Using the first two modeling methods, we sought to (i) identify cognitive diagnosis outcomes in this longitudinal prevalent PD cohort, (ii) determine biological factors related to cognitive diagnosis and dementia prediction, and (iii) establish any associations between genetic factors and specific cognitive test performance. Finally, using the multitask models, we sought to identify associations between cognitive test performance patterns and subsequent dementia.

## Results

### Overview

**Fig. 1:**
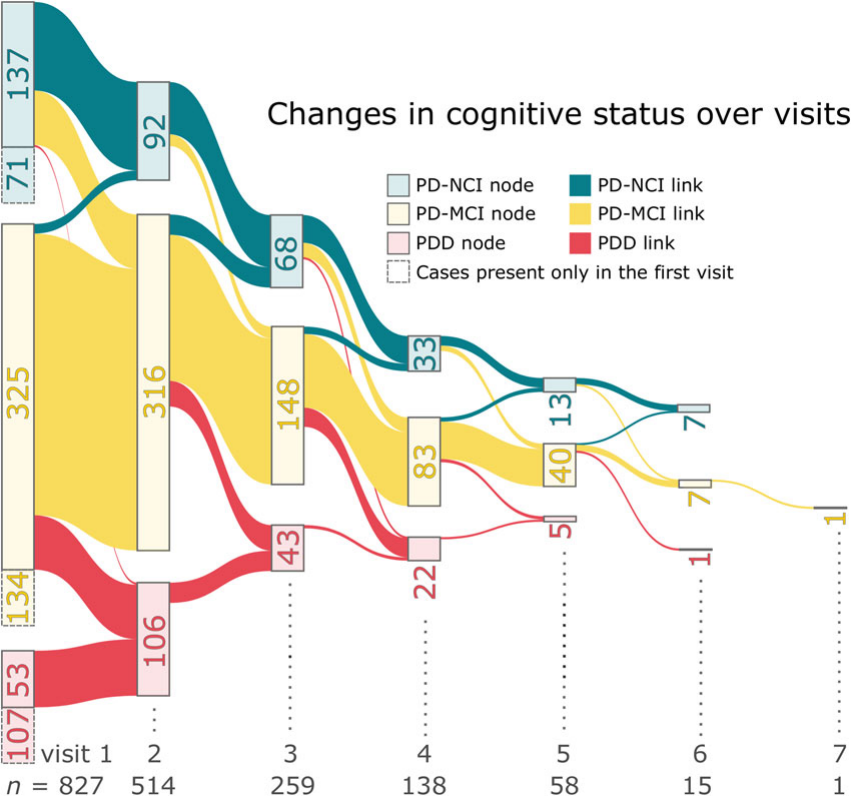
Changes in cognitive status across visits. The number inside each node represents the number of people with the corresponding cognitive status indicated by its color. The nodes with dashed line represent people with only data from the first visit. The links represents the group participants who continued to the next visit.

Fixed-effect, mixed-effect, and multitask learning models were implemented to analyze detailed cognitive and biological data from 827 participants with PD (514 with longitudinal data) enrolled in the PUC. Age, education, sex, disease duration (time since initial onset of PD motor symptoms), total levodopa equivalent daily dose (LEDD; calculated as described by Tomlinson et al.^6^), the 15-item Geriatric Depression Scale (GDS-15)^7^, and site were the included covariates. To determine whether the inclusion of younger participants influenced the results, analyses were repeated both for the entire sample and excluding participants under 50. Given that there were not substantial differences noted in the results, the following results are presented using the entire study sample. Baseline cohort characteristics are provided in Table 1. Longitudinal change in cognitive status (no cognitive impairment [NCI], mild cognitive impairment [PD-MCI], dementia [PDD]) across visits is depicted in Fig. 1.

**Table 1:** Baseline characteristics of the Pacific Udall Center cohort.

	NCI *n* = 208	PD-MCI *n* = 459	PDD *n* = 160	Overall *P*^a^ pairwise
Age at visit, years				
Mean (sd)	64.4 (8.3)	68.1 (8.9)	72.7 (9.3)	<0.0001
Range	40.3–83.9	36.2–90.1	48.9–91.8	NCI < PD-MCI/PDD, PD-MCI < PDD
Education, years				
Mean (sd)	16.3 (2.4)	15.9 (2.5)	15.4 (2.8)	0.002
Range	12–20	8–20	8–20	NCI > PDD
Sex				
* n* (%) male	97 (46.6%)	330 (71.9%)	140 (87.5%)	<0.0001 NCI < PD-MCI/PDD, PD-MCI < PDD
Disease duration, years				
Mean (sd)	7.6 (5.2)	8.7 (6.3)	11.9 (7.7)	<0.0001
Range	0–30	0–41	1–43	NCI < PDD, PD-MCI < PDD
Length of follow-up, years (*n* = 514)				
Mean (sd)	4.0 (2.1)	3.7 (2.0)	2.7 (1.3)	<0.0001
Range	1–8	1–8	1–7	NCI < PDD, PD-MCI < PDD
MDS-UPDRS				
Mean (sd)	21.0 (10.4)	27.0 (12.1)	36.3 (14.0)	<0.0001
Range	3–64	0–66	5–87	NCI < PD-MCI/PDD, PD-MCI < PDD
Modified Hoehn & Yahr				
Median	2	2	2.5	0.0001
Range	(1–4)	(1–5)	(1–5)	NCI < PD-MCI/PDD, PD-MCI < PDD
GDS-15				
Mean (sd)	5.4 (1.4)	5.9 (1.7)	6.8 (1.9)	<0.0001
Range	1–11	2–13	4–12	NCI < PD-MCI/PDD, PD-MCI < PDD
LEDD				
Mean (sd)	511.6 (455.2)	613.9 (497.6)	769.5 (560.2)	<0.0001
Range	0–2792	0–3375	0–3156	NCI < PDD, PD-MCI < PDD
*APOE*				
* n* (%) ε4 allele	43 (21.4%)	107 (23.7%)	32 (20.7%)	0.659
*GBA*				<0.001
* n* (%) carrier	15 (7.5%)	44 (9.7%)	34 (21.8%)	NCI < PDD, PD-MCI < PDD
*MAPT*				
* n* (%) H1 haplotype	50 (37.3%)	132 (33.5%)	42 (30.0%)	0.44
MoCA				
Mean (sd)	27.5 (1.9)	24.6 (2.5)	19.2 (4.3)	<0.0001
Range	22–30	17–30	7–29	NCI > PD-MCI/PDD, PD- MCI > PDD
HVLT-R total recall				
Mean (sd)	27.3 (3.5)	21.1 (4.6)	14.2 (4.5)	<0.0001
Range	15–35	7–34	5–30	NCI > PD-MCI/PDD, PD-MCI > PDD
HVLT-R delayed recall				
Mean (sd)	9.9 (2.1)	6.6 (3.2)	3.1 (2.9)	<0.0001
Range	0–12	0–12	0–11	NCI > PD-MCI/PDD, PD- MCI > PDD
HVLT-R RDI				
Mean (sd)	10.9 (1.2)	9.3 (2.2)	7.2 (2.5)	<0.0001
Range	3–12	0–12	−2–12	NCI > PD-MCI/PDD, PD-MCI > PDD
Trailmaking Test, Part A^b^				
Mean (sd)	29.1 (12.7)	40.4 (20.0)	72.6 (36.7)	<0.0001
Range	13–130	15–150	23–150	NCI < PD-MCI/PDD, PD-MCI < PDD
Trailmaking Test, Part B^b^				
Mean (sd)	67.9 (31.6)	120.7 (64.9)	238.8 (75.1)	<0.0001
Range	28–300	29–300	70–300	NCI < PD-MCI/PDD, PD-MCI < PDD
Trailmaking Part B – Part A^b^				
Mean (sd)	38.8 (27.2)	80.1 (56.7)	166.9 (65.8)	<0.0001
Range	2–272	7–272	30–264	NCI < PD-MCI/PDD, PD-MCI < PDD
Digit symbol				
Mean (sd)	50.6 (10.2)	38.9 (10.5)	24.2 (10.7)	<0.0001
Range	18–82	2–70	0–54	NCI > PD-MCI/PDD, PD-MCI > PDD
Letter number sequencing				
Mean (sd)	11.0 (2.4)	8.7 (2.4)	5.6 (2.5)	<0.0001
Range	3–18	0–18	0–12	NCI > PD-MCI/PDD, PD-MCI > PDD
Phonemic verbal fluency				
Mean (sd)	48.3 (11.7)	38.6 (11.5)	26.9 (9.9)	<0.0001
Range	22–93	8–84	8–53	NCI > PD-MCI/PDD, PD-MCI > PDD
Semantic verbal fluency				
Mean (sd)	23.3 (4.9)	18.5 (5.2)	11.6 (4.6)	<0.0001
Range	13–37	5–37	1–22	NCI > PD-MCI/PDD, PD-MCI > PDD
Judgment of line orientation				
Mean (sd)	13.0 (1.8)	11.8 (2.4)	9.8 (3.2)	<0.0001
Range	6–15	0–15	0–15	NCI > PD-MCI/PDD, PD-MCI > PDD

*APOE* apolipoprotein E gene, *GBA* glucocerebrosidase gene, *GDS-15* 15-item Geriatric Depression Scale, *HVLT-R* Hopkins Verbal Learning Test-Revised, *LEDD* levodopa equivalent daily dose, *MAPT* microtubule-associated protein tau gene, *MDS-UPDRS* Unified Parkinson’s Disease Rating Scale, Movement Disorders Society revision, *MoCA* Montreal Cognitive Assessment, *NCI* not cognitively impaired, *PDD* Parkinson’s disease dementia, *PD-MCI* Parkinson’s disease mild cognitive impairment, *RDI* recognition discriminability index, *sd* standard deviation.

^a^Overall (pairwise) comparisons based on one-way ANOVA (Scheffe’s test) for continuous variables, Kruskal–Wallis (Dunn’s test) for ordinal variables, or chi-square for dichotomous variables.

^b^Lower score = better performance.

### Effects of biological factors on cognitive status

**Fig. 2:**
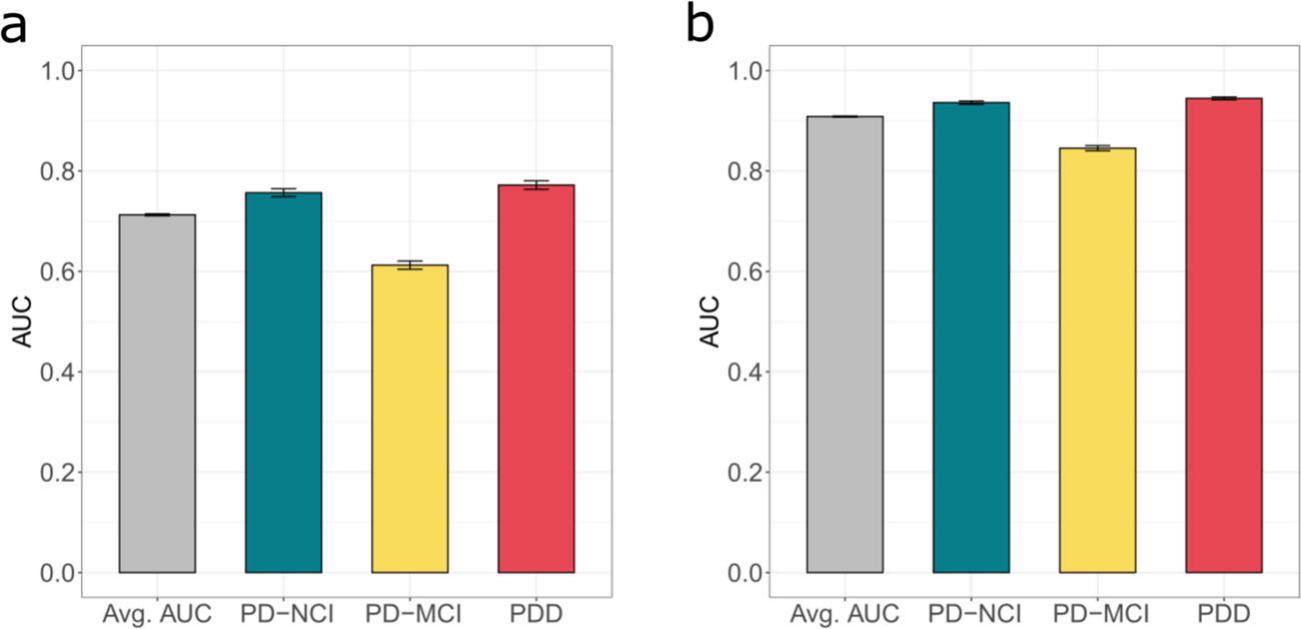
Biological factors satisfactorily predict cognitive status. Cross-validated area under receiver operating characteristic (AUC) of the mixed-effect model prediction based only on biological factors (**a**) compared to the AUC of the mixed-effect model prediction based solely on cognitive tests (**b**). Error bars represent standard deviations (sd).

In the entire sample, a mixed-effect model developed using only biological factors was found to have satisfactory prediction of cognitive status across all visits (average area under the receiver operating characteristic curve [AUC] = 0.71, Fig. 2a). Predictions of both PD-NCI and PDD (AUC = 0.76 and 0.77, respectively) were more accurate than PD-MCI (AUC = 0.61). Of note, this model using only biological factors performed worse than the model using only cognitive test performance (a major component in making a cognitive diagnosis) (average AUC = 0.9; Fig. 2b). In the final model, which included all covariates, all biological factors were significantly associated with cognitive status except for microtubule-associated protein tau (*MAPT)* and apolipoprotein E (*APOE)* genotype (Table 2). Notably, the increase in odds ratios of both being male and having a glucocerebrosidase gene (*GBA)* variant were approximately equivalent to an additional 15 years of PD duration in terms of PDD risk in this cohort.

**Table 2:** Association of biological factors with cognitive status in the full longitudinal PUC cohort.

	Coef. (OR)^a^	95% CI (OR)		*P*
Age	0.18 (1.20)	0.15 (1.16)	0.22 (1.24)	2.64 × 10^−26^
Disease duration	0.15 (1.16)	0.11 (1.12)	0.19 (1.21)	5.13 × 10^−14^
Sex	2.92 (18.54)	2.30 (9.97)	3.54 (34.47)	2.80 × 10^−20^
*APOE*	0.23 (1.26)	−0.39 (0.68)	0.85 (2.34)	0.47
*GBA*	2.72 (15.18)	1.94 (6.96)	3.50 (33.11)	1.00 × 10^−11^
*MAPT*	−0.21 (0.81)	−0.80 (0.45)	0.38 (1.46)	0.48

*APOE* apolipoprotein E gene, *CI* confidence interval, *GBA* glucocerebrosidase gene, *GDS-15* 15-item Geriatric Depression Scale, *LEDD* levodopa equivalent daily dose, *MAPT* microtubule-associated protein tau gene, *OR* odds ratios, *PUC* Pacific Udall Center.

^a^Models adjusted for LEDD, GDS-15, site and years of education.

**Fig. 3:**
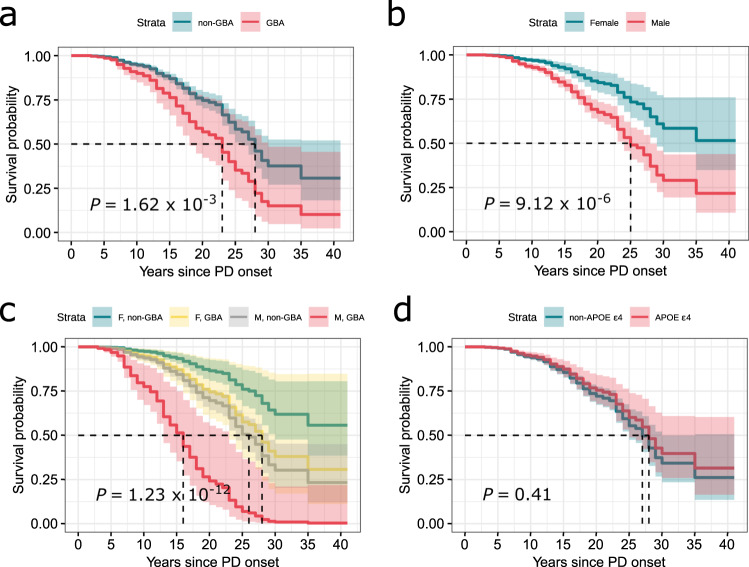
Survival analyses indicate significant longitudinal differences between participants of different sex and selected genes. Survival analyses to an endpoint of PDD for participants categorized by *GBA* variant (**a**), sex (**b**), combination of both (**c**), and *APOE ε4* allele (**d**) by the number of years since the diagnosis of PD. *P* value obtained from log rank tests indicated significant effect of sex, *GBA* variant, and the combination of both.

In the longitudinal cohort (excluding participants with PDD at baseline), survival analyses showed a significantly shorter duration between PD symptom onset and diagnosis of PDD in *GBA* mutation carriers compared to non-mutation carriers (Fig. 3a). Faster progression to PDD was also observed in males compared to females (Fig. 3b). Male participants with a *GBA* variant were starkly more at risk of acquiring PDD, and earlier, than female participants with no *GBA* variant (Fig. 3c). *APOE ε*4 did not exhibit a significant effect on time to PDD (Fig. 3d). The significance of these observations remains unchanged even if the time scale was changed to age at visit or to months since the first visit (Supplementary Fig. 1).

In analyses that were restricted to participants with longitudinal data who were nondemented at their first visit but were diagnosed with PDD at any subsequent visit (*n* = 97), age at PD onset was also a significant factor in the rate of progression to PDD (Supplementary Table 1). Number of years from PD onset until PDD and age at PDD are shown in Supplementary Fig. 2; no correlation was noted (*R* ≪ 0.1, results not shown). Later PD symptom onset was associated with faster progression to PDD (Supplementary Fig. 2).

### Effects of genetic factors on cognitive test performance

Analysis of the fitted mixed-effect model indicated the strongest effect on individual cognitive tests was from *GBA*, which was significantly associated with all tests except phonemic verbal fluency and Hopkins Verbal Learning Test-Revised (HVLT-R) Delayed Recall after Bonferroni correction (Table 3). Both *APOE* and *MAPT* did not exhibit significant effects after correction. However, analysis using a sex-specific cohort (females only) suggested a significant effect of *APOE ε*4 with lower performance on semantic verbal fluency (Supplementary Table 2). In addition, *GBA* effects on visuospatial and verbal learning tasks could be sex-specific (Supplementary Table 3). It should be noted that a generalizable predictive model could not be developed for this purpose due to large random effects between individuals (as evidenced by the relatively large standard errors of the random intercept for each test; Table 3).

**Table 3:** Association of *APOE ε*4 allele, *GBA* status, and *MAPT* haplotype with the cognitive performance in the full longitudinal PUC cohort.

Cognitive tests	Intercept	*APOE*				*GBA*				*MAPT*			
	Random Intercept Std.	Coef.^a^	95% CI	*P*	*P*_c_	Coef.^a^	95% CI	*P*	*P*_c_	Coef.^a^	95% CI	*P*	*P*_c_
Semantic verbal fluency	4.06	−1.11	−1.91 to −0.31	0.01	0.07	−1.83	−2.92 to −0.74	1.10 × 10^−3^	0.01	0.10	−0.66 to 0.85	0.80	>0.99
Phonemic verbal fluency	9.47	−1.46	−3.2 to 0.29	0.10	>0.99	−1.50	−3.87 to 0.86	0.22	>.99	−0.05	−1.7 to 1.6	0.95	>0.99
HVLT-R Total Recall	3.76	−0.38	−1.11 to 0.36	0.32	>0.99	−1.92	−2.92 to −0.92	1.84 × 10^−4^	1.84 × 10^−3^	0.58	−0.11 to 1.27	0.10	>0.99
HVLT-R RDI	1.30	0.00	−0.28 to 0.28	0.99	>0.99	−0.56	−0.94 to −0.18	4.12 × 10^−3^	0.04	0.11	−0.15 to 0.38	0.39	>0.99
HVLT-R Delayed recall	1.97	−0.07	−0.5 to 0.36	0.75	>0.99	−0.67	−1.25 to −0.08	0.03	0.27	0.36	−0.05 to 0.76	0.08	0.84
Judgment of line orientation	1.67	−0.44	−0.77 to −0.1	0.01	0.11	−0.99	−1.45 to −0.54	2.34 × 10^−5^	2.34 × 10^−4^	0.12	−0.19 to 0.44	0.45	>0.99
Digit symbol	8.97	−1.63	−3.29 to 0.03	0.06	0.56	−4.85	−7.1 to −2.6	2.98 × 10^−5^	2.98 × 10^−4^	0.27	−1.3 to 1.84	0.74	>0.99
Letter number sequencing	1.73	−0.11	−0.46 to 0.23	0.53	>0.99	−0.95	−1.42 to −0.48	9.28 × 10^−5^	9.28 × 10^−4^	0.35	0.02 to 0.67	0.04	0.37
TMT B – TMTA	40.27	4.50	−3.73 to 12.75	0.29	>0.99	31.47	20.21 to 42.72	6.74 × 10^−8^	6.74 × 10^−7^	−8.30	−16.09 to −0.53	0.04	0.32
MoCA	2.53	−0.37	−0.87 to 0.13	0.15	>0.99	−1.75	−2.43 to −1.07	7.24 × 10^−7^	7.24 × 10^−6^	0.20	−0.27 to 0.68	0.40	>0.99

*P*_c_ = Bonferroni corrected values.

*APOE*, apolipoprotein E gene, *CI* confidence interval, *GBA* glucocerebrosidase gene, *GDS-15* 15-item Geriatric Depression Scale, *HVLT-R* Hopkins Verbal Learning Test-Revised, *LEDD* levodopa equivalent daily dose, *MAPT* microtubule-associated protein tau gene, *MoCA* Montreal Cognitive Assessment, *PUC* Pacific Udall Center, *RDI* Recognition Discrimination Index, *TMT* Trailmaking Test.

^a^Models adjusted for age, LEDD, GDS-15, disease duration, sex, site and years of education.

### Prediction of future cognitive diagnosis by cognitive test performance

**Fig. 4:**
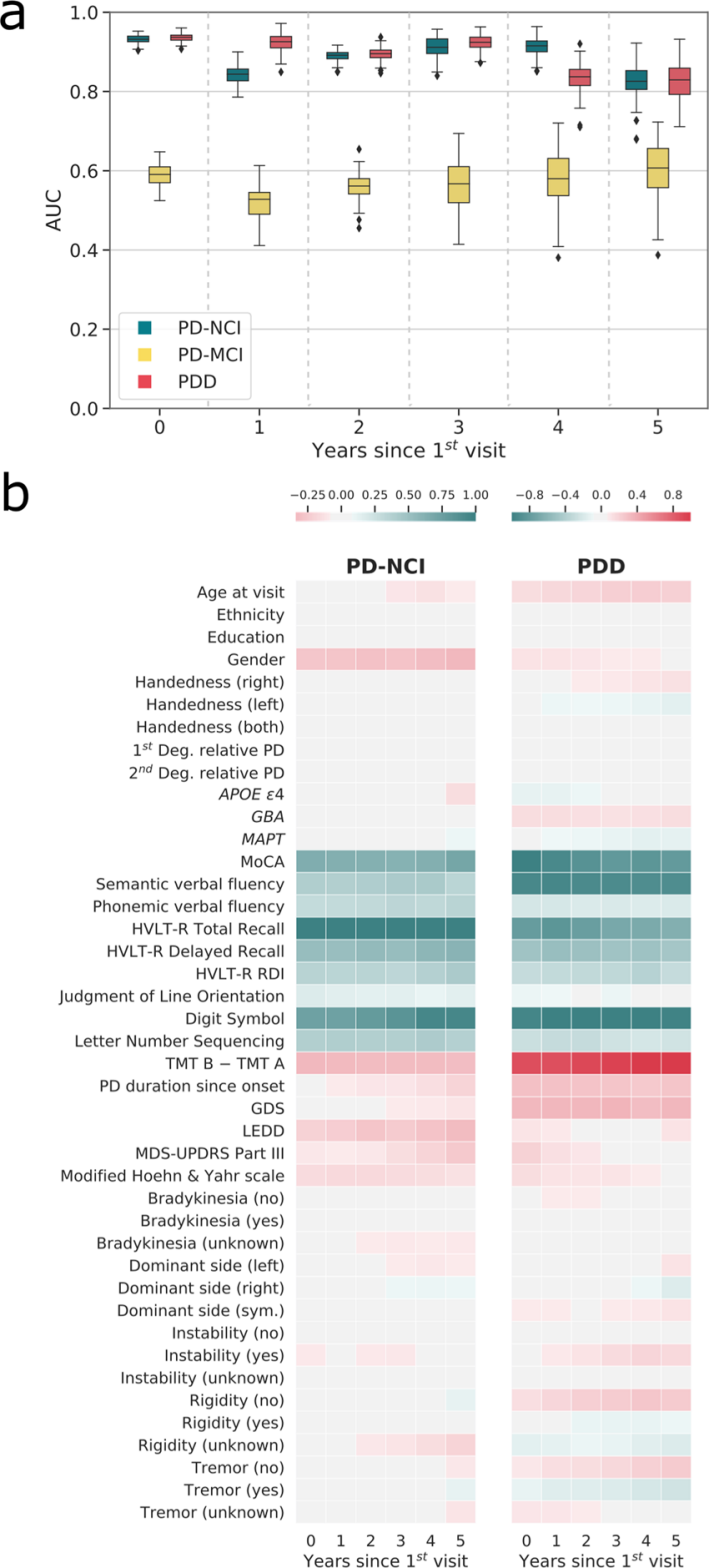
Multitask model indicates current test performances could imply future cognitive status. The area under receiver operating characteristic curve (AUC) of the multitask model prediction on unseen data with each task predicting the participants’ cognitive status at *n*^th^ years after the first visit using only their first visit and biological data (**a**). The median (Q2), the first and third quantile (Q1 and Q3), and the minimum and maximums (Q1−1.5IQR and Q3 + 1.5IQR) are at the center line, bounds, and the whiskers of the box plots. The heatmap depicting the magnitude of components from PD-NCI and PDD classification models, highlighting the importance of many of the cognitive tests in the prediction of future cognitive status. The positive components in each model are associated with higher probability of that model’s diagnosis (**b**).

Multitask models were employed for future cognitive status prediction, where each task predicted cognitive status of a specific year in the future based only on the data from the first visit (limited to five years since the first visit due to reduced numbers of visits beyond this point). The model could accurately separate PD-NCI from PDD up to four years into the future (Fig. 4a). However, the model could not accurately differentiate PD-MCI from other diagnoses in any year. Analysis of the model components indicated that cognitive tests are the most important features in the prediction of future cognitive status. Specifically, HVLT-R Total Recall and Digit Symbol scores were the most indicative of PD-NCI, whereas the Montreal Cognitive Assessment (MoCA), semantic verbal fluency, Digit Symbol, and Trailmaking Test B minus Trailmaking Test A (TMT B-A) were the most indicative of PDD (Fig. 4b). Other factors including sex, *GBA* status, and PD duration and severity also affected some tasks at a lower scale. This suggests that although biological factors are significant, cognitive test scores are stronger predictors of subsequent dementia. This is consistent with the mixed-effect analysis above which demonstrated that cognitive status is more strongly associated with combined cognitive test performance than the combination of biological factors at each visit (Fig. 2).

## Discussion

In the current study, we evaluated features related to patterns of cognitive progression in a large PD cohort. Age, disease duration, sex, and *GBA* status were the primary biological factors associated with cognitive status. Survival analyses demonstrated the importance of sex, *GBA*, and age of PD onset in the progression to PDD in this prevalent cohort. *GBA* carriers had worse performance across most cognitive measures, and potential sex-specific differences on specific cognitive tasks were noted in relation to *APOE* and *GBA*. Importantly, when all variables were included in the model, we found that although performance on specific cognitive tests best predicted subsequent cognitive status in the cohort for PD-NCI and PDD, this model could not accurately predict future PD-MCI.

The size of the PUC cohort, breadth of data collected, and longitudinal design permitted implementation of robust multivariate approaches to address important questions related to cognitive progression in people with PD. Increasingly, such methods are employed across disciplines to address shortcomings associated with traditional statistical approaches. While to date the use of machine learning approaches is limited in PD research, such methods have been used to predict disease progression in the Michael J Fox Foundation Parkinson’s Progression Markers Initiative (PPMI)^8^. Only one recent study included cognitive outcome in the PPMI cohort, and found that initial MoCA score, sleep symptoms, auditory working memory, and anxiety symptoms were the primary factors related to subsequent worsening global cognition. Unlike the current study, age, sex, and disease duration were not related to subsequent decline in global cognition^9^. However, PPMI enrolls participants with de novo PD, thus participants were only evaluated during the earliest stages of the disease when cognitive decline may be minimal. Further, the sample size was smaller and length of follow-up shorter than in the current analyses. Importantly, neuropsychological testing in this study included only the MoCA, compared to the depth and breadth of testing available in our cohort. Finally, genetic factors that may directly influence PD phenotypes were not included. For example, *GBA* variants have been associated with the above traits (anxiety^10^, auditory working memory^11^, sleep symptoms^12^). These phenotypic features may thus serve as a proxy for certain underlying biological traits in some participants. In the current study, we clearly demonstrate the important role of *GBA* in cognitive presentation and progression in PD, consistent with a previous longitudinal study by our group using traditional statistical methods^13^.

Although we, and now several others, have reported increased cross-sectional risk for dementia in people with PD who inherited an *APOE ε*4 allele^14–16^, our results here showed only a trend to an increased rate of progression to dementia in this group. These results mirror those for AD, where *APOE ε*4 is a strong and extensively replicated genetic risk factor; however, the impact of *APOE ε*4 on clinical progression to MCI or AD dementia in multivariate analyses is not clear. Indeed, some reported a significant impact of *APOE ε*4 on clinical progression to MCI or AD dementia, while others did not^17–20^. These studies show that the impact of *APOE ε*4 on clinical progression is complex, and several observed significant interactions with being female. Our results most closely match those from the Alzheimer’s Disease Neuroimaging Initiative, Australian Imaging, Biomarker and Lifestyle Study, and Harvard Aging Brain Study, which showed that *APOE ε*4 itself is not a major factor in clinical progression^18^. Although not a strong predictor of progression to PDD in our cohort, inheritance of an *APOE ε*4 allele was not benign; women with PD who had an *APOE ε*4 allele were at greater risk for decline in semantic verbal fluency. As we have previously shown, reduced semantic verbal fluency is associated with shortened time to PDD among females only^21^. In the AD literature, impaired semantic verbal fluency is associated with dementia diagnosis as well as with AD biomarkers in preclinical disease^22,23^, and there is some evidence that females with AD dementia may perform worse than males on semantic verbal fluency tasks^24^. Further, *APOE ε*4 may play a role in influencing semantic verbal fluency performance in amnestic mild cognitive impairment^25^. Taken together, these results tentatively suggest that *APOE ε*4 may have a greater impact on cognitive phenotype in females with PDD, although additional research is necessary. Finally, it is important to consider cohort characteristics among these many observational studies that may underlie some of the apparent discrepancies. Indeed, our cohort likely has under-sampled early PDD and this may undermine our ability to associate progression to PDD with *APOE ε*4. With this limitation in mind, our longitudinal results from people with PD align with most results from AD and highlight a possible but weak effect on the rate of progression to dementia, possible domain-specific effects, and potentially stronger impact on women.

Consistent with our previous cross-sectional reports^26,27^, we also found no association between the *MAPT* H1 haplotype and specific cognitive test performance, dementia diagnosis, or cognitive decline during follow-up. Previous reports on *MAPT* and cognition are mixed, with one group reporting faster decline in MMSE scores and greater dementia risk in PD patients with the H1 haplotype^28^ and another showing a greater association between the H1 haplotype and PD diagnosis among those with dementia^29^. However, many others have shown no association between cognitive test performance, cognitive diagnosis, or rate of cognitive decline and the H1 haplotype, and the current study provides additional evidence that the *MAPT* H1 haplotype may not play a primary role in cognitive decline in PD^30–32^.

The results from the current study extend our understanding of sex differences and cognitive decline in PD, particularly in association with genetic profile. As we and others have shown, male sex is associated with a higher likelihood of cognitive impairment and with faster progression of cognitive symptoms in PD^21^. Here, we demonstrate an additive relationship for *GBA* and sex in influencing the rate of progression to dementia, such that male *GBA* carriers progressed most quickly, while female *GBA* carriers had a similar rate of progression to that of male non-*GBA* carriers. Predictably, *GBA* carrier status was associated with worse performance in multiple domains for both males and females (global function, divided attention, working memory, and processing speed)^11,33^. However, while the previously reported association between *GBA* and lower visuospatial function in PD is replicated, in secondary analyses the association was only significant for males. Reduced visuospatial function has been implicated in conversion to dementia in PD^34,35^. Performance on the Judgment of Line Orientation task is most frequently correlated with lesions in the right posterior parietal-occipital regions^36^, areas where *GBA* carriers have demonstrated reduced synaptic activity and nigrostriatal DAT density^37^. Thus, the greater degree of cognitive decline in males with PD may be in part related to *GBA*-influenced lesions in these regions or in the pathways that serve these regions. Additional work in this area is needed to determine if *GBA* influences lesion location in brain differentially for males and females.

Overall, our multivariate approach showed that the prediction of placement into the cognitively unimpaired and PDD groups is quite high using all available variables, particularly specific cognitive measures. Our models could not, however, accurately predict PD-MCI. The identification of meaningful cognitive subtypes in PD-MCI has proven difficult given the heterogeneity of the disease^38^. Variability in PD-MCI is common, with a 24% average rate of reversion over 1–6 years of follow-up reported in a recent meta-analysis^2^. Medication effects, motor subtypes, anxiety, depression, fluctuations in attention, hallucination, delusions, and myriad other disease-related factors may impact cognitive function for those on the path to PDD, leading to diagnostic instability and difficulty predicting rate of cognitive decline^2^.

The primary limitation of the current study was that, due to enrollment of participants with prevalent PD, we were unable to follow the natural history of cognitive impairment from disease onset to dementia. As a result, those diagnosed with PDD early in the disease are likely under-sampled, leading to an inflated time to dementia when compared to what others have reported^39–41^. However, the goal of the current study was not to provide expected annual incidence rates of PDD, as these have been well-described previously. Rather, the goal was to identify important biological and cognitive factors that predict cognitive diagnosis; by enrolling a prevalent sample we were able to study the full cognitive diagnostic range even cross-sectionally at the initial visits, something that is not possible in an incident PD cohort^42^. Thus, although we provide survival analysis models to demonstrate the differences in time to PDD according to various biological factors, the absolute time values should not be taken to represent time to incident PDD in the entire PD population. Possible additional contributors to this finding of longer time to PDD in the cohort include (a) our measurement of disease onset from first motor symptoms vs. time of PD diagnosis, and (b) a substantially larger cohort than the previously mentioned studies, potentially leading to wider variability in PD phenotype. Future results from incident studies including larger samples will be informative. Further sampling limitations of the study include that our participants were generally highly educated, and thus may not be representative of the larger population with PD. Finally, due to the limitations of the data collected, we were not able to include potentially important variables in the analyses, such as the possible mediating effects of antidepressants and sedatives, vascular risk, and detailed sleep and anxiety features.

Cognitive impairment in PD is pervasive and distressing, and identification of factors associated with cognitive decline in PD may allow earlier intervention. Traditional statistical methods aimed at the identification of factors associated with cognitive progression may produce biased or spurious results. Our robust multivariate approaches to data collected from a large sample of participants with prevalent PD and varying levels of cognitive function reveal that the primary biological factors associated with PDD are male sex, *GBA* status, age, and disease duration, while performance on tasks measuring executive functions, semantic verbal fluency, and recall were the best predictors of subsequent PDD. PD-MCI was much more unstable and difficult to predict with either biological or cognitive variables. These results provide clinicians with data to aid in the identification of risk for PDD, and thus to implement important behavioral, social, and cognitive interventions to maximize quality of life in people with PD. Future work to better identify predictors of variability versus stability for those with PD-MCI will be important in the ongoing pursuit of optimally characterizing and introducing effective interventions for this sizable group of cognitively impaired individuals with PD.

## Methods

### Participants

Participants were enrolled in the PUC, a Morris K. Udall Center of Excellence in Parkinson’s Disease Research, which collects detailed longitudinal data from three sites: Stanford University, University of Washington/Veterans Affairs Puget Sound Health Care System, and Oregon Health Sciences University/Veterans Affairs Portland Health Care System. All participants met the United Kingdom Parkinson’s Disease Society Brain Bank diagnostic criteria for PD (UKPDBB); atypical parkinsonism syndromes were excluded. Participants were excluded from these analyses if they met UKPDBB criteria at their initial visit but did not meet criteria by their final visit and/or were determined to have parkinsonism related to other factors, or if there was not enough information to determine UKPDBB status (*n* = 19). Participants with an unknown/other cognitive diagnosis (*n* = 4) or those who were diagnosed with PDD but later reverted to PD-NCI or PD-MCI (*n* = 5; unexpected events likely due to factors such as anxiety, depression, illness, or medication effects) were excluded. There were no exclusions based on age at visit or age at symptom onset. Participants from all sites who completed at least one visit and who were assigned a cognitive diagnosis of PD-NCI, PD-MCI, or PDD were included (*n* = 827). Longitudinal analyses included participants with at least one follow-up examination (*n* = 514). Time between follow-up visits for most participants was 1–2 years; a smaller proportion had longer intervals (Supplementary Fig. 3).

### Ethical compliance

The institutional review boards at Stanford University, University of Washington/Veterans Affairs Puget Sound Health Care System, and Oregon Health Sciences University/Veterans Affairs Portland Health Care System provided formal approval for the study procedures. All participants (or a legally authorized representative) provided written informed consent.

### Consensus diagnosis

Participants were assigned motor and cognitive diagnoses during diagnostic consensus conferences attended by at least two movement disorders specialists and a neuropsychologist. Cognitive diagnoses were made according to published criteria^43,44^ as previously described^45^, and were based on data from neuropsychological testing (Supplementary Table 4) (comparing raw test scores to demographically corrected normative values), participant history, and clinical interview.

### Cognitive variables

The core cognitive variables included in the current analyses are those common to all sites: (1) global (MoCA^46^); (2) learning & memory (HVLT-R^47^); (3) attention/working memory (Letter-Number Sequencing from the Wechsler Adult Intelligence Scale – III^48^, Digit Symbol subtest from the Wechsler Adult Intelligence Scale-Revised^49^, Trailmaking Test, parts A and B^50^); (4) verbal fluency (animals and letters F-A-S^50^); and (5) visuospatial (Benton Judgment of Line Orientation^51^). Trailmaking Test B - A scores were used to minimize the effects of motor disability. Participants completed additional neuropsychological tests at each site to permit cognitive diagnosis using Movement Disorders Society PD-MCI Level II criteria (Supplementary Table 4). Raw test scores were used for the purposes of the analyses. Analyses including z-scores based on comparison to demographically corrected normative values were run separately; given that these did not produce substantially different results as compared to the raw scores, the results are not shown.

### Clinical variables and covariates

A movement disorder specialist assessed severity of motor symptoms using Part III of the Movement Disorder Society revision of the Unified Parkinson Disease Rating Scale (MDS-UPDRS)^52^ and the Modified Hoehn and Yahr scale^53^. Age, education, sex, disease duration (time since initial onset of PD motor symptoms), total LEDD, and GDS-15 were included as covariates. Site differences were seen at baseline with regard to education, motor severity, and cognitive severity/status (Supplementary Table 5), and thus site was also included as a covariate. Differences in time between visits for participants was accounted for by including age in all analytic models.

### Genetic variables

Genomic DNA was extracted from peripheral blood or saliva samples using standard methods. Participants were genotyped for *APOE* rs429358 and rs7412 (which define the *ε*2, *ε*3, and *ε*4 alleles) and *MAPT* rs1800547 (which differentiates H1 and H2 haplotypes) using commercially available assays TaqMan assays (Applied Biosystems)^27^. *APOE* genotype was encoded as either having at least one *ε*4 allele or none. Sequencing of the entire *GBA* coding region was performed to detect the presence of all known pathogenic mutations and the E326K polymorphism (rs2230288). “Pathogenic” mutations were defined as previously described^11^. *GBA* mutations and the E326K polymorphism were combined as a single group in dominant model analyses given our previous demonstration that both are associated with a higher risk of dementia and specific cognitive impairments^11,13^.

### Data preprocessing

Missing data points (2% of the total observed features) were imputed using Restricted Boltzmann machine.

### Linear fixed-effect and mixed-effect models

Ordinal mixed-effect regression with logit link^54^ were used to study the longitudinal association between biological factors and cognitive status. A linear mixed-effect regression^55^ was used to study the longitudinal association between biological factors and cognitive test performance. For both analyses, random intercepts were used to account for correlation within a participant. To examine the model performance in predicting cognitive status, the distribution of the reported AUC for each diagnosis (PD-NCI, PD-MCI, and PDD) was obtained from 100 iterations of two-layered cross-validations; in each iteration 25% of the data were held out for testing the model performance as unseen data and the inner cross-validation layer used the rest of the data for model fitting and optimization. While the prediction performance is objectively evaluated via cross-validation, a final model was fit and interpreted based on the entire data set, with potential confounders included as covariates. The two-sided *P* values from Wald tests of the coefficients were reported. For analysis of the progression rate based only on biological factors (cross-sectional data), a simple linear fixed-effect regression model was used.

### Generalized multitask models

Multitask models were used to predict future cognitive status based on data from the year of the first visit, i.e., each of the tasks predicted cognitive status for *n* (0–5) years in the future. Multitask learning aims to improve the generalization performance by exploiting the intrinsic relatedness and learning multiple related tasks simultaneously. A specific type of multitask learning, temporal grouped LASSO (TGL)^56^, was employed. With logistic loss, the TGL cost function is shown below as Eq. (1)

1 $$\min \mathop {\sum }\limits_{i = 1}^t \mathop {\sum }\limits_{j = 1}^{n_i} {\log} \left( {1 + \exp \left( { - Y_{i,j}\left( {W_i^TX_{i,j} + c_i} \right)} \right)} \right) \,+\, {\theta_1}\Vert W\Vert_F^2 + {\theta _2}\Vert WH \Vert_F^2 + {\theta _3}\Vert W \Vert_{2,1}$$

where ${{X}}_{i,j}$ denotes sample ${j}$ of the ${i}^{th}$ task, ${Y}_{i,j}$ is the corresponding ground truth of the sample, *W*_*i*_ and *c*_*i*_ are the model weights and biases for task ${i}$, ${\theta}_1$, ${\theta}_2$, and ${\theta}_3$ are regularization parameters controlling $\ell _2$-norm penalty, temporal smoothness, and group sparsity for joint feature selection, respectively (optimized during cross-validation), ${\mathrm{H}}$ is a matrix of temporal smoothness prior, where ${H} \in {\Bbb R}^{{t} \times ({t} - 1)}$ and ${H}_{ij} = 1$ if ${i} = {j},\,{H}_{ij}$ = −1 if ${i} = {j} + 1$, and ${H}_{ij}$ = 0 otherwise, $||.||_{\mathrm{F}}$ represents a Frobenius norm, and $||.||_{2,1}$ is $\mathop {\sum }\nolimits_{{i} = 1}^{d} {\mathrm{sqrt}}(\mathop {\sum }\nolimits_{{j} = 1}^{t} (.)_{{ij}}^2)$. Therefore, the first term measures empirical error of the model, the second penalizes overfitting (by penalizing large weights), the third term encourages temporal smooth transition (by penalizing large weight differences in the subsequent visit), i.e. assuming that most decline from PD-NCI to PDD transitions through PD-MCI, and the last term promotes the model to select the a feature subset from all ${\mathrm{d}}$ features that is important over all ${\mathrm{t}}$ tasks (by penalizing features that are not strong in all tasks). Through this knowledge sharing between tasks, TGL has previously shown superior performance for prognosis prediction compared to traditional machine learning algorithms^57^.

The TGL model was implemented in MATLAB through Malsar package^58^. The model, originally built for binary classification, was modified to handle ordinal classes according to a published protocol^59^. Specifically, two sparse regression models were built: one predicted the probability of PD-NCI and the other predicted the probability of PDD; the probability of PD-MCI was calculated as one minus these two predicted probabilities. The distribution of AUCs (PD-MCI vs. others; PDD vs. others) based on predicted probabilities across validations was obtained using a cross-validation scheme similar to the mixed-effect regression models. The final model was obtained by averaging the weights from all models predicting the probability of either being a certain cognitive status or not across cross-validation iterations.

### Survival analyses

Diagnosis of PDD was used as the endpoint in survival analyses. A Cox proportional hazards (Cox PH) regression with frailty, a type of mixed-effect survival model, was used to study the association between baseline covariates and time to PDD. The model was clustered by participants to account for correlated groups of observations and the log-rank test was performed to obtain the two-sided *P* value for each covariate. The survival curve in different subgroups was then generated using the fitted Cox PH model.

### Reporting summary

Further information on research design is available in the Nature Research Reporting Summary linked to this article.

## Supplementary information

Supplement

Reporting summary

## Data Availability

The data that support the findings of this study and scripts for data analysis are available from the corresponding author upon reasonable request. The data are not publicly available due to them containing information that could compromise research participant privacy consent. Data use agreements between the University of Washington/Dr. Zabetian and each outside investigator and their institutions are required. Such agreements would need to be completed by the researcher (and their institution) and the University of Washington prior to the raw data being made available.

## Code availability

The custom code used for analysis of the data was written in Python, R, and MATLAB languages. Python was used in data visualization, data cleaning and preprocessing, interface with MATLAB, and noSQL result storage. Multi-task learning was done using MATLAB. R was used for survival analysis and multi-level modeling. In survival analysis, the core packages used include survival and survminer. In multilevel modeling, the core packages used include lmerTest, vcrpart, and stats. The source code for reproducing the results shown in this study can be found at: https://github.com/tpjoe/Predicting-Dementia-in-Parkinson-s-Disease-Patients.
