# Supplementary material for: Multivariate prediction of dementia in Parkinson’s disease
Source: NPJ Parkinsons Dis. 2020 Aug 25;6:20. doi: 10.1038/s41531-020-00121-2 (PMC7447766; doi:10.1038/s41531-020-00121-2)
Supplement: Supplementary file 1 — Supplement [file 41531_2020_121_MOESM1_ESM.pdf]

Multivariate prediction of dementia in Parkinson's disease

Supplement

Supplementary Table 1. Association of biological factors with the disease progression rate to dementia (number of years from PD motor onset until PDD)

	Coef.*	95% CI		*P*
**Age at onset**	-0.37	-0.46	-0.27	7.02×10^{-11}
**Sex**	-1.85	-4.31	0.61	0.14
APOE	-2.08	-4.44	0.29	0.09
GBA	-5.42	-9.35	-1.49	8.23×10^{-3}
MAPT	-0.73	-2.97	1.52	0.53

*The model is adjusted for site and years of education. Only patients who do not have dementia at the first visit and are known to eventually be diagnosed with dementia are included (*n* = 97).

Abbreviations: *APOE*, apolipoprotein E gene; CI, confidence interval; *GBA*, glucocerebrosidase gene; *MAPT*, microtubule-associated protein tau gene

Supplementary Table 2. Association of *APOE* ε4 allele, *GBA* status, and *MAPT* haplotype with the cognitive performance in the longitudinal female PUC cohort.

Cognitive tests	Intercept	*APOE*				*GBA*				*MAPT*			
	Random Intercept Std.	**Coef.**	**95% CI**	***P***	P_c	**Coef.**	**95% CI**	***P***	P_c	**Coef.**	**95% CI**	***P***	P_c
**Semantic verbal fluency**	4.00	-2.45	-3.82 to -1.07	6.88×10^{-4}	6.88×10^{-3}	-2.37	-4.09 to -0.66	8.02×10^{-3}	0.08	0.18	-1.1 to 1.46	0.79	>.99
**Phonemic verbal fluency**	9.21	-3.48	-6.57 to -0.39	0.03	0.30	-2.74	-6.59 to 1.12	0.17	>.99	0.59	-2.29 to 3.48	0.69	>.99
**HVLT-R Total Recall**	3.57	-0.56	-1.82 to 0.7	0.39	>.99	-1.21	-2.78 to 0.36	0.14	>.99	0.48	-0.69 to 1.65	0.43	>.99
**HVLT-R RDI**	0.99	-0.30	-0.69 to 0.1	0.15	>.99	-0.49	-0.98 to 0.01	0.06	0.58	0.38	0.01 to 0.75	0.05	0.49
**HVLT-R Delayed Recall**	1.84	-0.21	-0.92 to 0.5	0.56	>.99	-0.70	-1.59 to 0.19	0.13	>.99	0.60	-0.06 to 1.27	0.08	0.80
**Judgment of Line Orientation**	1.51	-0.75	-1.32 to -0.18	0.12	0.11	-0.64	-1.35 to 0.07	0.08	0.83	0.13	-0.39 to 0.66	0.63	>.99
**Digit Symbol**	8.59	-3.72	-6.56 to -0.87	0.01	0.12	-4.74	-8.29 to -1.19	0.01	0.10	1.05	-1.6 to 3.71	0.44	>.99
**Letter Number Sequencing**	1.67	-0.43	-1.03 to 0.16	0.16	>.99	-1.14	-1.89 to -0.39	3.49×10^{-3}	0.03	0.21	-0.35 to 0.77	0.47	>.99
**TMT B – TMT A**	33.27	10.01	-2.83 to 22.87	0.13	>.99	29.03	12.94 to 45.14	6.07×10^{-4}	6.07×10^{-3}	-6.65	-18.71 to 5.27	0.28	>.99
**MoCA**	2.12	-0.58	-1.38 to 0.21	0.16	>.99	-1.74	-2.73 to -0.75	8.04×10^{-4}	8.04×10^{-3}	0.29	-0.45 to 1.03	0.45	>.99

*Adjusted for age, LEDD, GDS-15, disease duration, site and years of education.

P_c = Bonferroni corrected values

Abbreviations: *APOE*, apolipoprotein E gene; CI, confidence interval; *GBA*, glucocerebrosidase gene; GDS-15, 15-item Geriatric Depression Scale; HVLT-R, Hopkins Verbal Learning Test-Revised; LEDD, levodopa equivalent daily dose; *MAPT*, microtubule-associated protein tau gene; MoCA, Montreal Cognitive Assessment; PUC, Pacific Udall Center; RDI, Recognition Discrimination Index; TMT, Trailmaking Test

Supplementary Table 3. Association of *APOE ε4* allele, *GBA* status, and *MAPT* haplotype with the cognitive performance in the longitudinal male PUC cohort.

Cognitive tests	Intercept	*APOE*				*GBA*				*MAPT*			
	Random Intercept Std.	**Coef.**	**95% CI**	***P***	P_c	**Coef.**	**95% CI**	***P***	P_c	**Coef.**	**95% CI**	***P***	P_c
**Semantic verbal fluency**	4.05	-0.50	-1.48 to 0.48	0.32	>.99	-1.47	-2.89 to -0.06	0.04	0.42	-0.04	-0.96 to 0.89	0.94	>.99
**Phonemic verbal fluency**	9.63	-0.58	-2.7 to 1.54	0.59	>.99	-0.93	-3.96 to 2.09	0.55	>.99	-0.40	-2.42 to 1.62	0.70	>.99
**HVLT-R Total Recall**	3.80	-0.40	-1.3 to 0.49	0.38	>.99	-2.35	-3.64 to -1.07	3.87×10^{-4}	3.87×10^{-3}	0.56	-0.29 to 1.41	0.20	>.99
**HVLT-R RDI**	1.42	0.14	-0.23 to 0.5	0.46	>.99	-0.62	-1.14 to -0.09	0.02	0.23	-0.01	-0.36 to 0.33	0.93	>.99
**HVLT-R Delayed Recall**	2.02	-0.05	-0.58 to 0.48	0.86	>.99	-0.61	-1.39 to 0.16	0.12	>.99	0.20	-0.3 to 0.7	0.44	>.99
**Judgement of Line Orientation**	1.73	-0.29	-0.7 to 0.12	0.17	>.99	-1.27	-1.86 to -0.68	3.49×10^{-5}	3.49×10^{-4}	0.09	-0.3 to 0.48	0.67	>.99
**Digit Symbol**	9.06	-0.81	-2.83 to 1.22	0.44	>.99	-4.84	-7.74 to -1.94	1.22×10^{-3}	0.01	-0.10	-2.03 to 1.83	0.92	>.99
**Letter Number Sequencing**	1.76	0.03	-0.39 to 0.45	0.89	>.99	-0.87	-1.48 to -0.26	5.88×10^{-3}	0.06	0.39	-0.02 to 0.79	0.06	0.62
**TMT B – TMT A**	43.59	2.60	-7.86 to 13.08	0.63	>.99	35.99	20.87 to 51.09	4.35×10^{-6}	4.35×10^{-5}	-9.34	-19.28 to 0.59	0.07	0.68
**MoCA**	2.71	-0.25	-0.88 to 0.39	0.44	>.99	-1.81	-2.72 to -0.89	1.34×10^{-4}	1.34×10^{-3}	0.14	-0.46 to 0.74	0.65	>.99

*Adjusted for age, LEDD, GDS-15, disease duration, site and years of education.

P_c = Bonferroni corrected values

Abbreviations: *APOE*, apolipoprotein E gene; CI, confidence interval; *GBA*, glucocerebrosidase gene; GDS-15, 15-item Geriatric Depression Scale; HVLT-R, Hopkins Verbal Learning Test-Revised; LEDD, levodopa equivalent daily dose; *MAPT*, microtubule-associated protein tau gene; MoCA, Montreal Cognitive Assessment; PUC, Pacific Udall Center; RDI, Recognition Discrimination Index; TMT, Trailmaking Test

Supplementary Table 4. Neuropsychological measures used for cognitive diagnosis across PUC sites

	Neuropsychological measures	
**Cognitive domain**	**University of Washington/Oregon Health and Sciences University**	**Stanford University**
**Memory**	Hopkins Verbal Learning Test-Revised [1]<br>Logical Memory I & II* [2]<br>Brief Visual Memory Test-Revised [4]	Hopkins Verbal Learning Test-Revised [1]<br>Craft Story Recall† [3]<br>Benson Complex Figure recall† [5]
**Visuospatial**	Judgment of Line Orientation [6]<br>Clock copy [7]<br>Brief Visual Memory Test-Revised copy [4]	Judgment of Line Orientation [6]<br>Clock copy [7]<br>Benson Complex Figure copy† [5]
**Language**	Boston Naming Test* [8]<br>Shipley Vocabulary [10]<br>Semantic verbal fluency [12]	Multilingual Naming Test† [9]<br>Wechsler Test of Adult Reading [11]<br>Semantic verbal fluency [12]
**Executive**	Clock Drawing Test [7]<br>Phonemic verbal fluency [12]	Clock Drawing Test [7]<br>Phonemic verbal fluency [12]
**Attention/Working Memory**	Trailmaking test, parts A & B [12]<br>Letter-Number Sequencing [13]<br>Digit Symbol* [14]<br>Digit Span* [14]<br>Stroop (Golden version)[15]	Trailmaking test, parts A & B [12]<br>Letter-Number Sequencing [13]<br>Digit Symbol* [14]<br>Number Span†<br>Stroop (Victoria version) [12]

* National Alzheimer Coordinating Center Uniform Data Set, version 1
† National Alzheimer Coordinating Center Uniform Data Set, version 2

[1] R.H.B. Benedict, D. Schretlen, L. Groninger, J. Brandt, The Hopkins Verbal Learning Test-Revised: Normative data and analysis of inter-form and inter-rater reliability., The Clinical Neuropsychologist 12 (1998) 43-55.
[2] D. Wechsler, Wechsler Memory Scale-Revised Manual, The Psychological Corporation, San Antonio, 1987.
[3] S. Craft, J. Newcomer, S. Kanne, S. Dagogo-Jack, P. Cryer, Y. Sheline, J. Luby, A. Dagogo-Jack, A. Alderson, Memory improvement following induced hyperinsulinemia in Alzheimer's disease, Neurobiol Aging 17(1) (1996) 123-30.
[4] R.H.B. Benedict, BVMT-R Manual, PAR, Odessa, FL, 1988.
[5] K.L. Possin, V.R. Laluz, O.Z. Alcantar, B.L. Miller, J.H. Kramer, Distinct neuroanatomical substrates and cognitive mechanisms of figure copy performance in Alzheimer's disease and behavioral variant frontotemporal dementia, Neuropsychologia 49(1) (2011) 43-8.
[6] A.L. Benton, A.B. Sivan, K. Hamsher, N.R. Varney, O. Spreen, Contributions to neuropsychological assessment: A clinical manual, Oxford University Press, New York, NY, 1994.
[7] I. Rouleau, D.P. Salmon, N. Butters, C. Kennedy, K. McGuire, Quantitative and qualitative analyses of clock drawings in Alzheimer's and Huntington's disease, Brain Cogn 18(1) (1992) 70-87.
[8] E. Kaplan, H. Goodglass, S. Weintraub, Boston Naming Test, Lea & Febiger, Philadelphia, PA, 1983.
[9] I. Ivanova, D.P. Salmon, T.H. Gollan, The multilingual naming test in Alzheimer's disease: clues to the origin of naming impairments, J Int Neuropsychol Soc 19(3) (2013) 272-83.
[10] W.C. Shipley, Shipley-2 Manual, Western Psychological Services, Los Angeles, CA, 2009.
[11] H.A. Holdnack, Wechsler Test of Adult Reading, The Psychological Corporation, San Antonio, TX, 2001.
[12] E. Strauss, E.M.S. Sherman, O. Spreen, A compendium of neuropsychological tests : administration, norms, and commentary, 3rd ed., Oxford University Press, Oxford ; New York, 2006.
[13] D. Wechsler, WAiS-III® Administration and Scoring Manual, The Psychological Corporation Harcourt Brace & Company, San Antonio, TX, 1997.
[14] D. Wechsler, Wechsler Adult Intelligence Scale-Revised manual, The Psychological Corporation, San Antonio, 1987.
[15] C.J. Golden, Stroop Color and Word Test: A Manual for Clinical and Experimental Uses, Stoelting, Chicago, IL, 1978.

Supplementary Table 5. Baseline characteristics of the Pacific Udall Center cohort by site

	Seattle n = 573	Portland n = 188	Stanford n = 66	Overall *P** pairwise
**Age at visit, years**				
Mean (sd)	67.8 (9.7)	68.7 (8.4)	68.9 (6.6)	
Range	36.2 – 91.8	41.2 – 90.2	50.7 – 83.4	0.378
**Education, years**				
Mean (sd)	15.8 (2.5)	15.7 (2.7)	17.2 (2.5)	<0.0001
Range	8 - 20	8 - 20	12 - 20	Stanford>Seattle/Portland
**Sex**				<0.0001
n (%) male	373 (65.1%)	159 (84.6%)	35 (53.0%)	Portland>Seattle/Stanford
**Disease duration, years**				
Mean (sd)	9.2 (6.6)	8.9 (6.5)	8.3 (5.9)	
Range	0 - 43	1 - 33	1 - 30	0.543
**PDD**				0.005
n (%)	115 (20.1)	42 (22.3)	3 (4.6)	Portland/Seattle>Stanford
**PD-MCI**				<0.001
n (%)	317 (55.3)	115 (61.2)	27 (40.9)	Portland>Seattle/Stanford, Seattle>Stanford
**MDS-UPDRS**				<0.0001
Mean (sd)	27.1 (12.8)	30.3 (14.1)	21.0 (10.1)	Portland>Seattle/Stanford,
Range	0 - 87	3 - 72	4 - 64	Seattle>Stanford
**Modified Hoehn & Yahr**				0.0001
Median	2	2.5	2	Portland>Seattle/Stanford,
Range	(1 – 5)	(1 – 5)	(1 – 4)	Seattle>Stanford
**GDS-15**				
Mean (sd)	6.0 (1.7)	5.9 (1.7)	5.5 (1.5)	
Range	1 - 13	2 - 11	2 - 12	0.076
**LEDD**				
Mean (sd)	622.3 (526.2)	692.5 (492.7)	377.7 (242.9)	0.0001
Range	0 - 3375	0 - 2876	0 - 1125	Stanford<Seattle/Portland
APOE				
n (%) ε4 allele	126 (22.8%)	45 (23.9%)	11 (16.7%)	0.465
GBA				
n (%) carrier	69 (12.3%)	15 (8.6%)	8 (12.7%)	0.274
MAPT				
n (%) H1 haplotype	174 (33.9%)	50 (32.5%)	-	0.750
**MoCA**				<0.0001
Mean (sd)	24.4 (3.8)	23.5 (4.1)	26.2 (3.7)	Stanford>Seattle/Portland,
Range	7 - 30	7 - 30	11 - 30	Seattle>Portland

* Overall (pairwise) comparisons based on one-way ANOVA (Scheffe's test) for continuous variables, Kruskal-Wallis (Dunn's test) for ordinal variables, or chi-square for dichotomous variables

[†] Lower score = better performance

Abbreviations: *APOE*, apolipoprotein E gene; *GBA*, glucocerebrosidase gene; GDS-15, 15-item Geriatric Depression Scale; HVLT-R, Hopkins Verbal Learning Test-Revised; LEDD, levodopa equivalent daily dose; *MAPT*, microtubule-associated protein tau gene; MDS-UPDRS, Unified Parkinson's Disease Rating Scale, Movement Disorders Society revision; MoCA, Montreal Cognitive Assessment; NCI, not cognitively impaired; PDD, Parkinson's disease dementia; PD-MCI, Parkinson's disease mild cognitive impairment; RDI, recognition discriminability index; sd, standard deviation

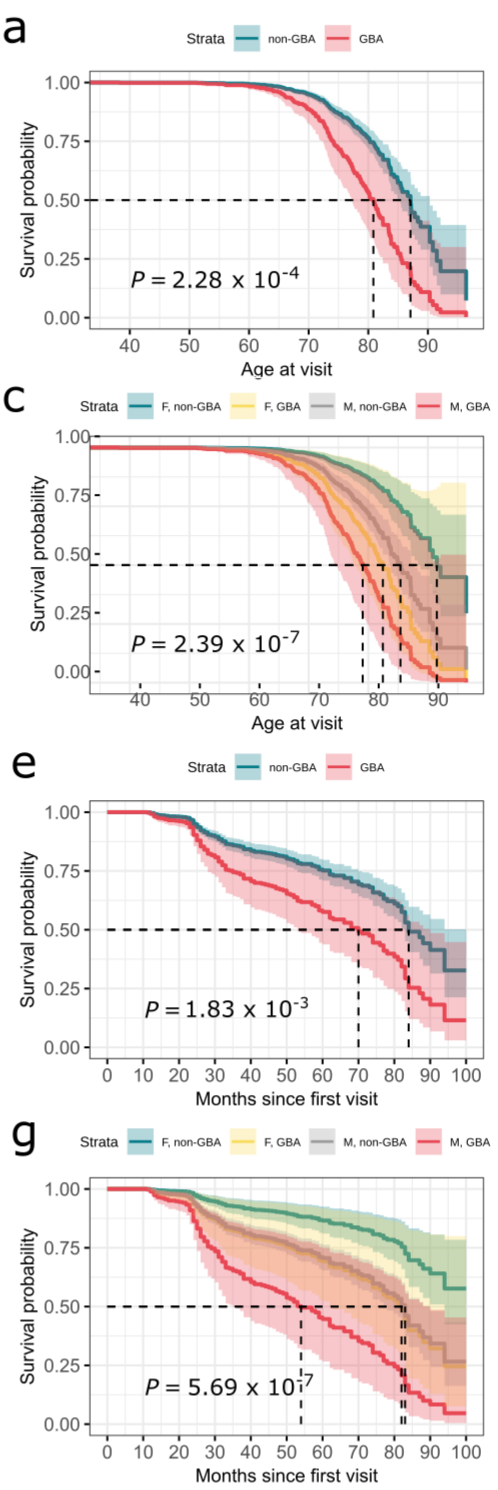

Supplementary Figure 1. Survival analyses indicate significant longitudinal differences between participants of different sex and selected genes. Survival analyses to an endpoint of dementia for participants categorized by GBA mutation (a, e), sex (b, f), combination of both (c, g), and *APOE ε4* allele (d, h) either by the age at their visit or months since their first visit. *P* value obtained from log ratio tests indicated significant effect of sex, *GBA* mutation, and the combination of both.

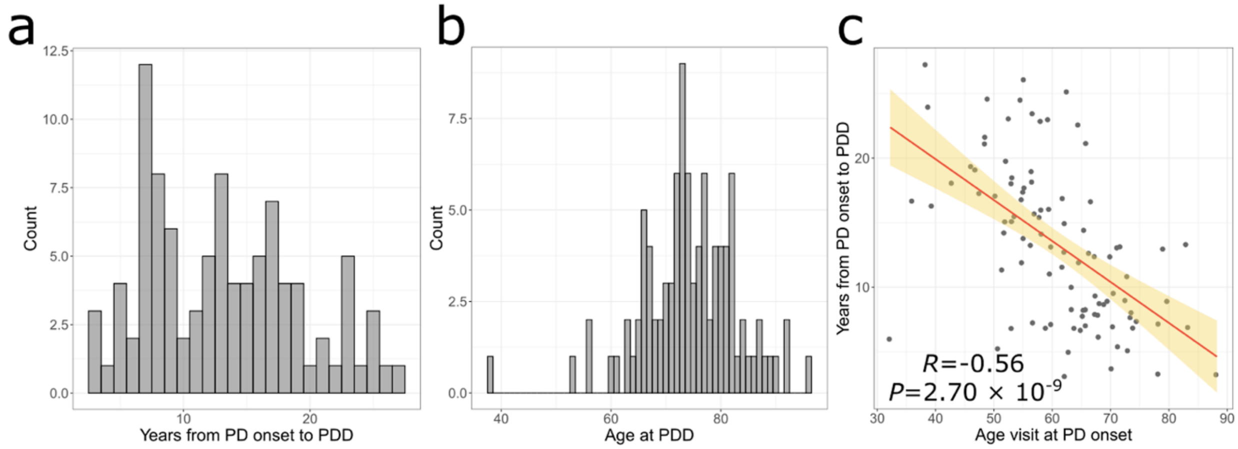

Supplementary Figure 2. The age at PD onset is a significant factor affecting progression rate to dementia. The distribution of the number of years until a person with PD was diagnosed with PDD (a) and the age at which they were diagnosed with PDD (b). The correlation between age at PD symptom onset to the number of years before they develop PDD (c). The analysis was restricted to patients with more than one time point, did not have dementia in the first visit, and was observed to develop dementia within the data set (n = 97).

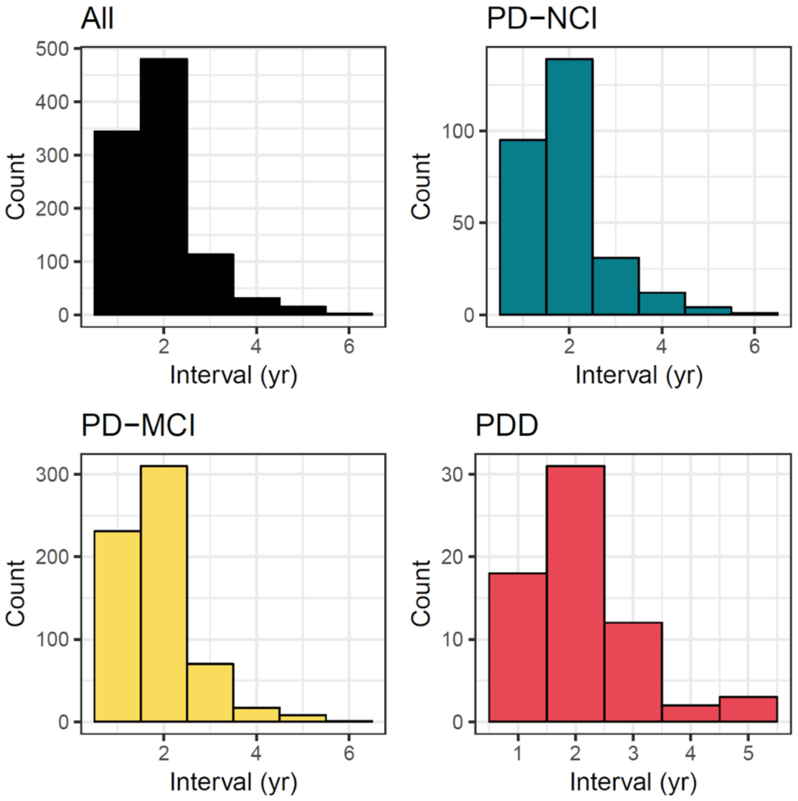

Supplementary Figure 3. Distribution of longitudinal visit intervals (in years) in the PUC
