# Supplementary material for: Multivariate prediction of dementia in Parkinson’s disease
Source: NPJ Parkinsons Dis. 2020 Aug 25;6:20. doi: 10.1038/s41531-020-00121-2 (PMC7447766; doi:10.1038/s41531-020-00121-2)
Supplement: Supplementary file 2 — Reporting summary [file 41531_2020_121_MOESM2_ESM.pdf]

nature research

Corresponding author(s): Brenna Cholerton, PhD

Last updated by author(s): Jul 13, 2020

# Reporting Summary

Nature Research wishes to improve the reproducibility of the work that we publish. This form provides structure for consistency and transparency in reporting. For further information on Nature Research policies, see Authors & Referees and the Editorial Policy Checklist.

## Statistics

For all statistical analyses, confirm that the following items are present in the figure legend, table legend, main text, or Methods section.

| n/a | Confirmed | |
|---|---|---|
| ☐ | ☒ | The exact sample size (*n*) for each experimental group/condition, given as a discrete number and unit of measurement |
| ☐ | ☒ | A statement on whether measurements were taken from distinct samples or whether the same sample was measured repeatedly |
| ☐ | ☒ | The statistical test(s) used AND whether they are one- or two-sided<br>*Only common tests should be described solely by name; describe more complex techniques in the Methods section.* |
| ☐ | ☒ | A description of all covariates tested |
| ☐ | ☒ | A description of any assumptions or corrections, such as tests of normality and adjustment for multiple comparisons |
| ☐ | ☒ | A full description of the statistical parameters including central tendency (e.g. means) or other basic estimates (e.g. regression coefficient) AND variation (e.g. standard deviation) or associated estimates of uncertainty (e.g. confidence intervals) |
| ☐ | ☒ | For null hypothesis testing, the test statistic (e.g. *F*, *t*, *r*) with confidence intervals, effect sizes, degrees of freedom and *P* value noted<br>*Give P values as exact values whenever suitable.* |
| ☒ | ☐ | For Bayesian analysis, information on the choice of priors and Markov chain Monte Carlo settings |
| ☒ | ☐ | For hierarchical and complex designs, identification of the appropriate level for tests and full reporting of outcomes |
| ☐ | ☒ | Estimates of effect sizes (e.g. Cohen's *d*, Pearson's *r*), indicating how they were calculated |

*Our web collection on statistics for biologists contains articles on many of the points above.*

## Software and code

Policy information about availability of computer code

| | |
|---|---|
| Data collection | Microsoft Access was used for data collection |
| Data analysis | The custom code used for analysis of the data was written in Python, R, and MATLAB languages. Python was used in data visualization, data cleaning and preprocessing, interface with MATLAB, and noSQL result storage. Multi-task learning was done using MATLAB. R was used for survival analysis and multi-level modeling. In survival analysis, the core packages used include survival and survminer. In multilevel modeling, the core packages used include lmerTest, vcrpart, and stats.The source code for reproducing the results shown in this study can be found at: https://github.com/tpjoe/Predicting-Dementia-in-Parkinson-s-Disease-Patients |

For manuscripts utilizing custom algorithms or software that are central to the research but not yet described in published literature, software must be made available to editors/reviewers. We strongly encourage code deposition in a community repository (e.g. GitHub). See the Nature Research guidelines for submitting code & software for further information.

## Data

Policy information about availability of data

All manuscripts must include a data availability statement. This statement should provide the following information, where applicable:

- Accession codes, unique identifiers, or web links for publicly available datasets
- A list of figures that have associated raw data
- A description of any restrictions on data availability

The data that support the findings of this study and scripts for data analysis are available from the corresponding author upon reasonable request. The data are not publicly available due to them containing information that could compromise research participant privacy consent. Data use agreements between the University of Washington/Dr. Zabetian and each outside investigator and their institutions are required.

# Field-specific reporting

Please select the one below that is the best fit for your research. If you are not sure, read the appropriate sections before making your selection.

☐ Life sciences ☒ Behavioural & social sciences ☐ Ecological, evolutionary & environmental sciences

For a reference copy of the document with all sections, see nature.com/documents/nr-reporting-summary-flat.pdf

# Behavioural & social sciences study design

All studies must disclose on these points even when the disclosure is negative.

| | |
|---|---|
| Study description | Cross-sectional and longitudinal quantitative study of participants with prevalent Parkinson's disease |
| Research sample | The sample consists of participants with a prevalent diagnosis of Parkinson's disease. It is a representative sample of the disease in that it includes participants with a wide range of motor and cognitive features at various stages of the disease. Gender breakdown is representative of Parkinson's disease (more males than females). The sample is highly educated and largely of European descent, which is consistent with the communities from which the participants were drawn but not representative of the population at large. |
| Sampling strategy | This is a convenience sample, recruited from local movement disorders clinics and a statewide Parkinson's disease registry for the purpose of enrolling a Clinical Core for the Pacific Udall Center, a Parkinson's Disease Research Center of Excellence. Sample size was based on the needs of the Core. |
| Data collection | Cognitive tests were administered by a research technician (trained by a neuropsychologist). A trained movement disorders specialist saw each participant in order to assess motor symptoms and to gather participant history. Additional forms administered to the participants and co-participants to assess depression, sleep quality, neuropsychiatric symptoms, and other pertinent history were administered by a research technician, study nurse, physician's assistant, or other trained study personnel. Blood was drawn by a trained phlebotomist. |
| Timing | Data collection began in 2010 and ended in February 2019 for the purposes of these analyses (enrollment and data collection for the Clinical Core is ongoing) |
| Data exclusions | 4 cases were excluded who had unknown/other cognitive status during one of their visits. Five participants who were diagnosed with dementia but later reverted to non-demented were excluded as these were regarded as rare and unexpected events |
| Non-participation | 62% of the included sample completed at least one follow up examination. Reasons for discontinuation included advanced dementia/ cognitive impairment, advanced motor disease, death, relocation, or inability to make contact at the time of follow up. |
| Randomization | Participants were not allocated to experimental groups |

# Reporting for specific materials, systems and methods

We require information from authors about some types of materials, experimental systems and methods used in many studies. Here, indicate whether each material, system or method listed is relevant to your study. If you are not sure if a list item applies to your research, read the appropriate section before selecting a response.

## Materials & experimental systems

| n/a | Involved in the study | |
|---|---|---|
| ☒ | ☐ | Antibodies |
| ☒ | ☐ | Eukaryotic cell lines |
| ☒ | ☐ | Palaeontology |
| ☒ | ☐ | Animals and other organisms |
| ☐ | ☒ | Human research participants |
| ☐ | ☒ | Clinical data |

## Methods

| n/a | Involved in the study | |
|---|---|---|
| ☒ | ☐ | ChIP-seq |
| ☒ | ☐ | Flow cytometry |
| ☒ | ☐ | MRI-based neuroimaging |

## Human research participants

Policy information about studies involving human research participants

| | |
|---|---|
| Population characteristics | Participants are adults, ages 36 - 91 diagnosed with Parkinson's disease with a mean of 16 years of education. 68% of the sample was male. |
| Recruitment | See above |

| | |
|---|---|
| Ethics oversight | Institutional review boards at the University of Washington,/VA Puget Sound Health Care System, Oregon Health and Sciences University/ VA Portland Health Care System, and Stanford University provided approval and oversight for this study |

Note that full information on the approval of the study protocol must also be provided in the manuscript.

# Clinical data

Policy information about clinical studies

All manuscripts should comply with the ICMJE guidelines for publication of clinical research and a completed CONSORT checklist must be included with all submissions.

| | |
|---|---|
| Clinical trial registration | N/A |
| Study protocol | This is not a clinical trial |
| Data collection | Recruitment and data collection began in 2010 and are ongoing for the Pacific Udall Center Clinical Core at three sites: University of Washington/VA Puget Sound Health Care System, Oregon Health and Sciences University/VA Portland Health Care System, and Stanford University |
| Outcomes | Primary outcome measures: cognitive performance at each visit; genetic data. Neuropsychological tests were administered and blood samples were collected as described above. |
